# Supplementary material for: Assessment of the Genetic Diversity and Population Structure of Rhizophora apiculata Blume (Rhizophoraceae) in Thailand
Source: Biology (Basel). 2022 Oct 1;11(10):1449. doi: 10.3390/biology11101449 (PMC9598538; doi:10.3390/biology11101449)
Supplement: Supplementary file 1 [file biology-11-01449-s001.zip › Figure S1.pdf]

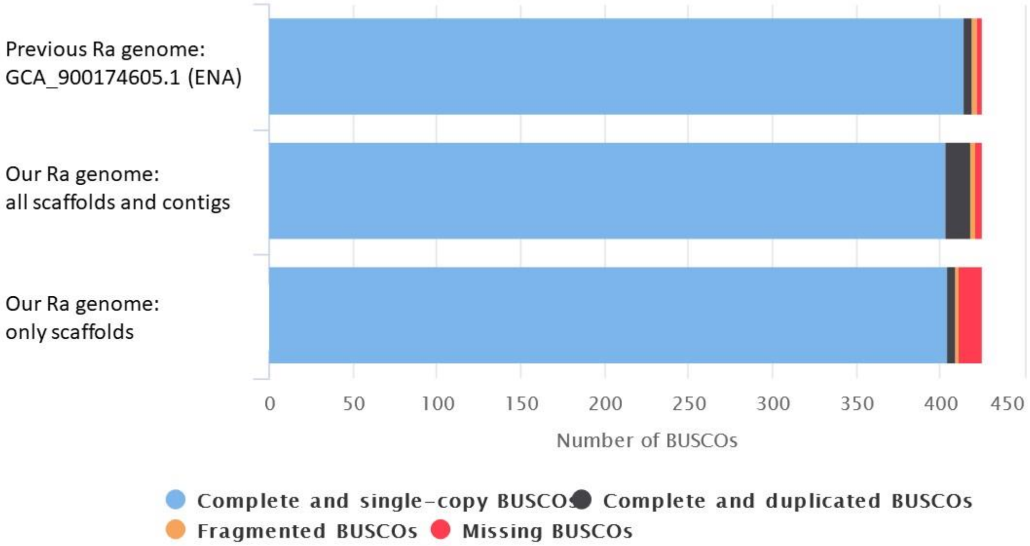

Figure S1 Genome assembly evaluation of *Rhizophora apiculata* using BUSCO. The datasets consist of the previous *R. apiculata* genome (GCA_900174605.1; ENA), our *R. apiculata* genome (133 scaffolds and 10,427 contigs), and the subset of our *R. apiculata* genome (133 scaffolds) (Table S1).
